# Rebuilding Earthquake Struck Nepal through Community Engagement

**DOI:** 10.3389/fpubh.2016.00121

**Published:** 2016-06-13

**Authors:** Bipin Adhikari, Shiva Raj Mishra, Shristi Raut

**Affiliations:** ^1^Mahidol-Oxford Tropical Medicine Research Unit, Faculty of Tropical Medicine, Mahidol University, Bangkok, Thailand; ^2^School of Population Health, University of Western Australia, Crawley, WA, Australia; ^3^Department of Microbiology, Lumbini Medical College, Palpa, Nepal

**Keywords:** earthquake, health education, health promotion, community engagement, Nepal

## Abstract

Nepal underwent two major earthquakes during 2015 which claimed 9,000 deaths, left more than 23,000 injured, displaced about 2 million people and destroyed about 1,000 health facilities. Emerging health issues and disease outbreaks soon after the earthquakes were major priorities. However, preventive measures such as health education, health promotion and trainings embedded in community engagement remained largely unimplemented. Establishing community preparedness by delivering knowledge about the disasters, preparing contingency plans and conducting disaster drills can be promising in Nepal where geographical inaccessibility invariably impedes the on time management during disasters. The steps that could be taken in Nepal without additional resources include identifying community leaders and volunteers who could participate in health promotion initiatives, training of thus identified community volunteers, formation of community task force, devolvement of responsibilities with continual support (trainings and resources) and supervision of the community task force.

## Earthquake and Possible Outbreak of Diseases

Disasters can affect all dimensions of human civilization, including damages to physical infrastructure and natural ecosystems (1). Nepal has been vulnerable to various types of disasters. Annually, Nepal experiences an average of nearly 300 natural disasters, such as lightning strikes, floods, earthquakes, and landslides. Between 1971 and 2012, over 28,000 people died from these natural disasters (2). On April 25, 2015 an earthquake measuring 7.8 on the Richter scale that ravaged Nepal affected 31 out of 75 districts (3, 4). Another earthquake that subsequently shook Nepal on May 12, 2015, caused further damage. These two earthquakes claimed 9,000 deaths, left more than 23,000 injured, and displaced about 2 million people. In addition, more than 500,000 houses and 1,000 health facilities were destroyed (5).

The burden of recovering (resettlement and renovation of functional infrastructure) from the damages had several challenges. Immediately after the earthquake, emerging health issues and disease outbreaks were the priority. Discussions on possible outbreak of infectious diseases after the earthquake divided the scientific communities (6, 7). Amidst the fear of impending disease, health-care workers advocated and recommended preemptive and/or reactive treatment plans (3, 4). Preventive measures, such as health education, community engagement, and health promotion, were promoted, however, remained largely unimplemented.

Evidences accumulated in recent years indicate that risk of infectious disease outbreaks after disasters are minimal, unless there is a displacement of population with poor water and sanitation conditions (5). In this particular situation, in addition to the immediate need for curative measures, consideration of sustainable preventable measures through health education and health promotion at the community level are imperative. Not only do health education and health promotion prevent possible disease outbreaks but they also increase the overall healthy behaviors and sustainable health literacy that can better prepare the population for the future (8).

## Health Education Amidst the Natural Disaster

Health education and promotion can bring positive changes in populations affected by disasters. The improved level of knowledge and consequent preparedness are far more promising than the “treat when sick” strategy. After the Ilapel earthquake, the Chilean approach of promoting calm was recognized as beneficial in preparedness and building resilience to cope more effectively with the incident (9). In New Zealand, the population was found to have higher resilience that was associated with health-related quality of life and well-being. This further emphasizes how intangible attributes of a population, such as emotional and spiritual well-being, could be conducive to preparedness for natural disasters, such as tsunami and earthquakes (10).

In Iran, health education and training were found to increase the overall ability for confrontation and readiness toward disaster situations, such as an earthquake (11). In addition to the benefits of preparedness for specific disaster situations, health education and promotion can deliver knowledge about disease prevention and improvements in hygiene and ongoing health practices. The after effects of health education and promotion in the Wenchuan, Chinese earthquake were remarkable as they increased the personal hygiene, health knowledge, and health practices from pre-intervention levels around 50% to post-intervention levels above 90% (8).

In addition to the immediate benefits, the sustainable effects of health education and promotion are far more promising. They include the empowerment of community members by increasing health literacy, risk reduction skills, the ability to advocate for health conducive behavior, and community capacity to future disasters. Nonetheless, health education and promotion have often been deprioritized and overlooked in preparing for and managing disasters.

## Prevention through Health Education in Nepal

As a result of the two earthquakes of 2015, more than 2 million people in Nepal have been displaced from their homes and have been obliged to live in makeshift shelter with compromised water and unsanitary conditions (12). In the month of August 2015, 20 cases of cholera were reported in an interval of less than a week in a single health facility in Kathmandu, the capital of Nepal. According to the Ministry of Health, 74% of water samples from the earthquakes affected areas were reported not suitable for drinking purposes (13). This was further compounded by the fact that 38% of households in these areas did not have toilet facilities (14). Fortunately, no major infectious disease outbreaks were reported in the country; however, elevated risks for future outbreaks are persistent because of the continuing lack of clean water and poor sanitation (15).

Seasonal epidemics (monsoon) and endemic food and water borne diseases make Nepal vulnerable in the future as well (15). While the measure taken to control epidemics, such as pre-positioning emergency supplies, is one mechanism of increasing preparedness for disasters, health promotion during, before, and after a disaster can influence the general health status of the population (16). However, delivering health education and health promotion in any context is a challenge. Without community being central to these activities, health education and promotion are always subjected to a potential gap between the provider and receiver. In a recent Ebola epidemic in West Africa, lack of community engagement and acceptance and heightened fear were realized to be the main reasons for failure of containment (17). Increasing evidence has accrued for many disease outbreaks in various parts of the world, such as cholera, dengue, shigellosis and diarrhea that their containment has been successful where health education and health promotion efforts have involved the community at the local level (18–21).

## Policies for Disaster Management and Community Engagement

The government of Nepal established the first structured disaster policy in 1982 with the Natural Calamity (Disaster) Relief Act and then expanded the legal framework with the local self-governance act in 1999. The National Strategy for Disaster Risk Management (NSDRM) Nepal 2009 provides the calculated guidance that encompasses all phases of the disaster management cycle (22). Despite the presence of clear plans and strategies for disaster mitigation by NSDRM (23), a recent study found that lack of coordination, unclear distribution of responsibility, and ineffective leadership at the policy level were barriers to efficient management during recent earthquakes (24). While NSDRM’s efforts in risk reduction during recent earthquake had limitations, yet another shortcoming in its policy is the lack of community level participatory approach, for which implementation is still far-fetched.

In Nepal, during and after the disasters, health education and health promotion activities apparently have been non-existent and community engagement has not yet been conceived by the government as essential. Given the status of disaster management at the national level, the vertical governance structure at the local level was bound to experience failure (24). Increasingly, in recent years, community participatory approaches to disaster management are recommended, ranging from the Ebola containment in West Africa (17, 25) to disaster preparedness management in Hong Kong (26).

The community engagement through sharing the leadership with the community has been proven to be most effective in health interventions in many African countries where not only the interventions were found effective (achieving desired health outcome), but the applied strategies were more economical than the conventional vertical approach (27).

A community-focused approach to inform, empower, and build the capacity for disaster preparedness and mitigation can be more effective than even the robust disaster plans of skilled workers. A disaster preparedness program where contingency plans and disaster drills have been carefully considered and rehearsed with the community can be learned from Hong Kong (26).

Establishing community preparedness by delivering knowledge about the disasters, preparing contingency plans, and conducting preemptive disaster drills can be highly effective in remediating disasters; and this is even more evident in a country like Nepal where geography and accessibilities are often factors that impede professionally trained internal and external experts from acting urgently during disaster situations (26). In addition, building community (local) capacity to deal with disaster situations can further fill the time lapse (time taken by government/disaster reduction agencies to reach the communities), while strengthening the coordination with disaster reduction strategies of government and non-government organizations.

## Conclusion

Nepal is vulnerable to various natural disasters. However, sustainable management through health promotion and health education through community engagement were largely neglected.

Current disaster plans of Nepal lack the community participatory approach for risk reduction and disaster management. An effective community engagement wherein involvement of the local community in planning, drills, health education, and mobilization can lead to effective control, resilience, and future preparedness for such disasters.

Nepal has laid a good legal and policy foundation with NSDRM. Now is the time to focus on implementation through community engagement. The elements of community engagement for health promotion are well documented from several field works where it has been successfully implemented (28). Steps that could be taken in Nepal with reallocation of existing resources, rather than additional resources, include

- identifying community leaders within the community who could participate in health promotion initiatives including disaster preparedness for future,
- forming a community task force (group of community volunteers) who can be trained with health education, health promotion, disaster preparedness, and emergency primary care,
- devolvement of responsibilities for the formed, trained community task force with the community’s consensus and initiative,
- monitoring the community directed approach from both local and national perspectives, including transparency in sharing performance data, and
- providing continual trainings and resources as and when necessary to maintain strong community preparedness designed to help to protect the community in the face of future disasters.

## Author Contributions

Exploration of the topic, review of literature, and manuscript write up. All authors listed, have made substantial, direct and intellectual contribution to the work, and approved it for publication.

## Conflict of Interest Statement

The authors declare that the research was conducted in the absence of any commercial or financial relationships that could be construed as a potential conflict of interest.
